# Clinical application of virtual reality in patients with cardiovascular disease: state of the art

**DOI:** 10.3389/fcvm.2024.1356361

**Published:** 2024-04-03

**Authors:** Valentina Micheluzzi, Eliano Pio Navarese, Pierluigi Merella, Giuseppe Talanas, Graziana Viola, Stefano Bandino, Chiara Idini, Francesco Burrai, Gavino Casu

**Affiliations:** ^1^ Clinical and Experimental Cardiology, Clinical and Interventional Cardiology, University Hospital, Sassari, Italy; ^2^ Department of Medicine, Surgery and Pharmacy, University of Sassari, Sassari, Italy

**Keywords:** virtual reality, atherosclerotic cardiovascular disease, cardiac rehabilitation, interventional cardiology, cardiac surgery

## Abstract

Virtual reality offers a multisensory experience to patients, allowing them to hear, watch, and interact in a virtual environment. Immersive virtual reality is particularly suitable for the purpose of completely isolating patients from the external environment to transport them away from the suffering related to the disease. On this state of the art, we summarize the available literature on the effectiveness of virtual reality on various physical and psychological outcomes in patients with atherosclerotic cardiovascular disease. Virtual reality has been employed in the cardiovascular field in various settings such as cardiac rehabilitation, interventional cardiology, and cardiac surgery. This technology offers promising opportunities to improve several outcomes related to cardiovascular disease, but further research is needed to entirely capture its benefits and to standardize the intervention.

## 1 Introduction

Despite the declining incidence and mortality rates of atherosclerotic cardiovascular disease (ASCVD) in numerous European nations, this disease remains a major cause of morbidity and mortality (1). Patients with ASCVD suffer from a plurality of physical and psychological symptoms that can significantly affect daily life activities (2). A notable psychological sequelae in this cohort is the apprehension regarding disease recurrence, which has been correlated with manifestations of posttraumatic stress disorder, prognostic deterioration, diminished wellbeing, and reduced quality of life (3). Additional psychological burdens include concerns about long-term survival, distress related to the initial diagnosis, trepidation about potential cardiac events, and uncertainty regarding clinical outcomes, all of which may precipitate symptoms of anxiety and depression (3). Moreover, hospitalization itself can be a source of psychological distress, often exacerbating recovery uncertainties and engendering feelings of helplessness. Anxiety is one of the most frequently observed psychological reactions among patients awaiting various surgeries and can occur in up to 80% of patients (4). An increased level of preoperative anxiety is related to both psychological and somatic negative consequences, which subsequently affects anesthesia administration, postoperative care, treatment, and the recovery process (4). Preoperative anxiety is also considered a risk factor for mortality in patients after surgery (4). Furthermore, the duration of procedures can increase acute psychological symptoms (5). For these reasons, improving psychological symptoms in patients with ASCVD is a crucial goal.

Virtual reality (VR) is an innovative technology that has been shown to improve outcomes related to ASCVD. VR is defined as the use of interactive simulations generated with computer hardware and software that allow patients to be physically and psychologically present in virtual environments and perceive real feelings, emotions, perceptions, and interactions (6). In this review, we describe the state-of-the-art effectiveness of VR applied to ASCVD patients in different settings.

## 2 The VR technology

VR can be divided into three distinct categories: non-immersive (nVR), semi-immersive (sVR), and immersive (iVR) (7). Non-immersive VR uses a screen that produces two-dimensional (2D) images, audio output, and joysticks for interaction, while semi-immersive VR also includes body sensors. Both non-immersive and semi-immersive VR can be distracting for people due to potential environmental perturbations. Immersive VR, conversely, offers high-quality three-dimensional (3D) images, delivered through an audio–visual head-mounted headset and controllers that allow full interaction with the virtual world (8).

The latest generation of iVR devices consists of a comfortable and lightweight head-mounted display (HMD) with high-performance processors onboard and a viewer composed of high-definition and high-pixel liquid crystal displays. The device also has a facial tracking sensor that enables 360° motion with clear, vivid colors and high-resolution images. At the audio level, iVR technology allows high-definition stereo, clear, natural sounds with a high dynamic range. At the tactile level, haptic gloves or sensory gloves can help patients feel the size, rigidity, and resistance of virtual objects, allowing them to sense holding, pushing, and touching these objects. This type of VR completely isolates patients from their physical external environment, enabling them to immerse themselves in the virtual experience through a multisensory approach (9). In sVR, users can interact with the virtual environment using controllers, but the images are reproduced on a 3D screen and not through an HMD, which provides better isolation. Comparing both iVR and sVR, iVR offers significantly improved virtual experiences because of increased computing power, the use of more sophisticated software, better optical components, low latency (the delay between action and reaction), a wide field of view, and increased interactivity. iVR has become a powerful distraction intervention for patients (10). It is an intervention used in healthcare to transport patients away from suffering, allowing them to perceive the virtual experience as real (11). Its use creates a more intense illusion of reality, bringing the individual's experience closer, more isolated, and more immersed in the multisensory environment. Beyond the classification into the three distinct categories of VR, another application choice of this technology can be made based on the type of selected content. In the cardiovascular field, as we will see in what follows, relaxing content, educational content, and recreational content defined as gaming apps have been used.

### 2.1 VR application in cardiac rehabilitation

In the field of cardiac rehabilitation (CR), three meta-analyses are presented in the literature. These include a total of 13 randomized controlled trials (RCTs) all using sVR with interactive contents such as gaming apps and that analyze different physical and psychological outcomes. A systematic review and meta-analysis conducted by Bashir et al. showed a significant decline in the levels of anxiety in four included patients, standard mean difference (SMD) = −0.32, 95% confidence interval (CI) −0.61 to −0.03, but no significant improvement in patients with functional capacity in ASCVD who underwent CR with sVR compared with standard CR (SMD = 0.077, 95% CI—1.24). However, the high degree of bias, the small sample size, and the considerable variability in exercise regimens could have influenced the results. Furthermore, the non-significance of the improvement in functional capacity can be explained by the fact that in the RCTs, the data were collected and measured at different times of CR (12). Chen et al. conducted a systematic review and meta-analysis including 10 RCTs. The results showed that VR applied to CR was able to increase exercise capacity, with mean difference (MD) = 49.55, 95% CI = 30.59–68.52, and reduce the following: stress (MD = −80, 95% CI = −1.17 to −0.43), depression (SMD = −0.48, 95% CI = −0.84 to −0.12), low-density lipoprotein concentration (MD = −1.79, 95% CI = −8.96 to −5.38), and total cholesterol (MD = −22.21, CI = −37.35 to −7.07). The evidence was statistically non-significant for anxiety, high-density lipoprotein, triglycerides, and body mass index (BMI). In contrast to the results indicating non-significance in triglyceride improvement, it is noteworthy that in the study by Vieira et al. (13) (the only study not in favor of VR included in the meta-analysis), 82% of patients in the VR group and 100% in the control group were on statin therapy, which acted as a confounding variable in addition to the small sample size of 20 patients (14).

A systematic review and meta-analysis by Blasco-Peris et al. showed no differences between VR and conventional CR programs for enhancing exercise capacity, with MD = 14.07 m (95% CI = 38.18–66.32 m, *p* = 0.426) and mental health [SMD = 0.17 (95% CI = 0.36–0.70; *p* = 0.358)]. The results showed a small, statistically non-significant improvement in quality of life in favor of exergaming [SMD = 0.22 (95% CI = −0.37 to 0.81)]. Moderate heterogeneity was found for exercise capacity (*I*^2^ = 53.7%), while no heterogeneity was found for quality of life (*I*^2^ = 3.3%) and mental health (*I*^2^ = 0.0%). These findings should be viewed with caution because of the low number of included studies, a high degree of RCT bias, and the non-homogeneity of the included sample (two studies recruited exclusively male patients and six included both male and female patients, but most of them were males) (15). The results of the three systematic reviews and meta-analysis are summarized in Table 1.

**Table 1:** Systematic review and meta-analysis in cardiac rehabilitation.

Meta-analyses	Setting	Intervention	Comparation	Studies (*N*)	Patients (*n*)	Results
The impact of virtual reality on anxiety and functional capacity in cardiac rehabilitation: a systematic review and meta-analysis (12)	CR home and center based	sVR with gaming apps’ contents	Standard CR	7 RCT	747	• Significant reduction of anxiety • No significant improvement in functional capacity
Effectiveness of virtual reality in cardiac rehabilitation: a systematic review and meta-analysis of randomized controlled trials (14)	CR home and center based	sVR with gaming app's contents	Standard CR	10 RCT	N/A	• Significant improvements include increased exercise capacity, reduced stress, decreased depression, and lowered levels of low-density lipoprotein and total cholesterol. • No significant results were observed in anxiety, high-density lipoprotein, triglycerides, and body mass index
Effects of exergaming in patients with cardiovascular disease compared to conventional cardiac rehabilitation: a systematic review and meta-analysis (15)	CR home or center based	sVR with gaming apps’ contents	Standard CR	8 RCT	733	• No significant improvement in exercise capacity, quality of life, and mental health

The effect of sVR was also investigated on cardiorespiratory parameters, and hemodynamic parameters were evaluated at both peak and submaximal exertions. The sVR group achieved a significantly higher value in maximum oxygen consumption (Vo2peak), peak metabolic equivalents (METS), and amount of Vo2 at the anaerobic threshold than the non-VR group (16). sVR also proved to be more effective in achieving the heart rate target of 85% of maximum heart rate and the metabolic cost target, while the control group did not reach the target or achieved it with a higher number of rehabilitation sessions (17). Because of a low number of RCTs, small sample size, low statistical power, a high degree of RCT bias, and moderate and high heterogeneity, it is very hard to generalize evidence in this field of care.

## 3 VR application in interventional cardiology and electrophysiology

iVR generates positive emotions and mood enhancements by synchronizing and stabilizing electronic impulses in the prefrontal brain, consequently reducing the patient's pain and anxiety (18, 19). Nevertheless, a few studies with very different applications and with variegated multimedia content have evaluated the effect of VR on interventional cardiology procedures.

A study by Keshvari et al. showed that 5 min of iVR with natural scenarios and sounds including soft music, birdsong, and waterfall sounds before invasive coronary angiography (ICA) helped reduce anxiety, as evaluated by the state trait anxiety inventory questionnaire (STAI), with significant differences with the standard care control group. The intervention was done only in the intervention group in a private silent room. Furthermore, they demonstrated that 5 min of iVR used before ICA helped improve heart rate and blood pressure (20).

A study by Aardoom et al. demonstrated that the use of educational content planned 1 or 2 weeks before the scheduled elective ICA could decrease anxiety related to the procedure in a better way.

Through iVR performed at home or in the hospital, patients could virtually explore the day of cardiac catheterization, including all phases from their hospital admission to the postprocedural period. During this experience, various topics regarding the care process were addressed, such as the activities in the ward, the necessary clothing, who could be at the patient's side, and the drugs administered after the operation. The patients could fully interact with avatars in the form of healthcare professionals to obtain relevant information at every stage. The entire virtual experience lasted approximately 20 min but probably varied depending on the time the patient spent in each module (21).

Another RCT by Morgan et al. showed a significantly greater reduction in anxiety in patients who used 10 min of iVR pre-ICA with specific educational content that illustrated the preprocedural phase and the procedure itself compared with patients in the conventional care group. The iVR group also had better procedural understanding and higher overall satisfaction than the control group (22). In the RCT by Gökçe and Arslan, the application of iVR to patients undergoing coronary angiography also proved to be more effective in reducing pain and anxiety scores and improving the patient’s comfort level, systolic blood pressure, respiratory rate, and pulse rate than traditional care provided in the control group (23).

iVR technology was also used in patients undergoing conscious sedation during the transfemoral transcatheter aortic valve replacement (TAVR) procedure by Bruno et al. The patients in the intervention group immersed themselves for a medium time of 30 min in relaxing virtual environments that they had previously selected. A total of 81.3% of the patients had used iVR until device implantation and 37.5% during the whole procedure. The results demonstrated a significant reduction in anxiety, measured with the visual Analog scale (VAS), compared with the control group patients who underwent standard TAVR. However, it is important to interpret these results with caution because of the small sample size (32 patients) used (24).

iVR has also been used in catheter ablation for atrial fibrillation (AF) before the start of the procedure with positive results in terms of patient knowledge, self-efficacy, greater satisfaction, and fewer concerns regarding the procedure (25–27). Furthermore, iVR before ablation is effective at reducing procedural anxiety and pain (27). Brewer et al. used iVR in 40 patients who underwent unilateral great saphenous vein radiofrequency ablation. All procedures were completed successfully with no difference reported in total surgery times, and all patients were generally satisfied with the treatment. Procedural anxiety was statistically significantly reduced by iVR compared with the standard care control group. Furthermore, anxiety in the control group patients increased during the procedure, while in the iVR group patients, it decreased. Finally, 85% of patients in the VR group would recommend the use of VR to someone undergoing a similar procedure (28). The results of RCTs in interventional cardiology are summarized in Table 2.

**Table 2:** RCTs in interventional cardiology and electrophysiology.

RCTs	Setting	Intervention	Comparation	Patients (*n*)	Results
The effect of virtual reality distraction on reducing patients’ anxiety before coronary angiography: a randomized clinical trial study (20)	Pre-ICA	iVR with relaxing scenarios	Standard care	60	• Reduction in anxiety • Improvement of heart rate and blood pressure
A preoperative virtual reality app for patients scheduled for cardiac catheterization: pre-post questionnaire study examining feasibility, usability, and acceptability (21)	Pre-ICA	iVR with educational content	Standard care	8	• Reduction in anxiety
The effect of a VIrtual RealiTy immersive experience upon anxiety levels, procedural understanding, and satisfaction in patients undergoing CArdiac CaTHeterization: the VIRTUAL CATH trial (22)	Pre-ICA	iVR with educational content	Standard care	64	• Reduction in anxiety • Better procedural understanding and higher satisfaction
Effects of virtual reality and acupressure interventions on pain, anxiety, vital signs and comfort in catheter extraction processes for patients undergoing coronary angiography: a randomized controlled trial (23)	Undergoing ICA	iVR with relaxing scenarios	Standard care	102	• Significant reduction of pain, comfort anxiety • Significant improvement of systolic blood pressure, respiratory rate, and heart rate
Virtual reality-assisted conscious sedation during transcatheter aortic valve implantation: a randomised pilot study (24)	Undergoing TAVR	iVR with relaxing scenarios	Standard care	32	• Significant reduction in anxiety
360° virtual reality to improve patient education and reduce anxiety towards atrial fibrillation ablation (25)	Pre-AF ablation	iVR with educational content	Standard care	134	• Positive results in terms of patient knowledge, greater satisfaction, and fewer concerns regarding the procedure
Virtual reality-based preprocedural education increases preparedness and satisfaction of patients about the catheter ablation of atrial fibrillation (26)	Pre-AF ablation	iVR with educational content	Standard care	32	• High level of satisfaction • Improve self-efficacy and knowledge
Virtual reality–based preprocedural education increases the preparedness and satisfaction of patients about the catheter ablation of atrial fibrillation (27)	Pre-AF ablation	iVR with specific educational content	Standard care	33	• Improve self-efficacy and knowledge • Reduce pain, anxiety, and impatience
Virtual reality can reduce anxiety during office-based great saphenous vein radiofrequency ablation (28)	Undergoing AF ablation	iVR with relaxing content	Standard care	40	• Reduction in anxiety

## 4 VR application in cardiac surgery

In the context of cardiac surgeries, patients often experience high levels of preoperative anxiety, particularly because of a lack of information about the impending procedure. Preoperative anxiety is influenced by various factors, including the patient's medical experience and information received prior to surgery. A study by Grab et al. explored the use of iVR patient education in the preoperative period, with the patient viewing 3D models of surgical procedures that illustrate the main steps of the surgery. Their results showed that VR with educational content improved patient understanding of surgery and reduced anxiety (29). Hendricks et al. compared the effectiveness of iVR with sVR in reducing preoperative anxiety in patients undergoing cardiac surgery. On the day of surgery, patients in the interventional group were allowed to use iVR and those in the control group were allowed to use an sVR through a game-type app for 20 min, the time typically spent waiting in the preoperative preparation area. The patients in the iVR group experienced a significant reduction in anxiety, which was measured with the STAI scale, compared with those in the sVR group. The use of iVR demonstrated significant benefits in patient experience, suggesting that it could be a promising option to reduce preoperative anxiety and improve patient wellbeing. VR usage might be considered an alternative to traditional pharmacological strategies to reduce preoperative anxiety, thereby improving overall patient experience during the preoperative period (30). For postoperative pain management, the use of iVR was compared with an inhaled equimolar mixture of N_2_O and O_2_ (Kalinox®) during removal of chest drains after cardiac surgery in intensive care patients. The analgesia/nociception index (ANI) was monitored to objectively evaluate pain and anxiety, and self-reported pain and anxiety data were collected using a numerical rating scale (NRS). The results showed that, according to the ANI, the iVR did not meet the statistical requirements to prove non-inferiority compared with Kalinox® in managing pain and anxiety during removal of chest drainage. Furthermore, patients in the iVR group reported significantly higher pain levels in the NRS immediately after drainage removal compared with those in the Kalinox® group, although there were no significant differences after 10 min. Therefore, VR was also found to be less effective according to the NRS (31). The effectiveness of iVR on anxiety, pain, fatigue and relaxation, physiological parameters, and opioid use in pre- and postoperative patients in cardiac surgery was also compared with a group that was subjected to hypnosis, a group that was subjected to both hypnosis and iVR, and a control group in standard care. The results showed that anxiety was significantly higher in the group of patients who underwent hypnosis compared with those in the group who were subjected to a combination of iVR and hypnosis. Pain decreased over time in all groups, with no significant differences between the groups. There were also no significant differences between the groups regarding opioid use. Overall, the techniques did not appear to have a significant impact on anxiety, pain, fatigue, and relaxation. Physiological parameters such as blood pressure, heart rate, and oxygen saturation were not influenced by the technique used (32).The results of RCTs in interventional cardiology and electrophysiology are summarized in Table 3.

**Table 3:** RCTs in cardiac surgery.

RCTs	Setting	Intervention	Comparation	Patients (*n*)	Results
New perspectives in patient education for cardiac surgery using 3D-printing and virtual reality (29)	Presurgery	iVR with educational content	Standard care	99	• Improved patient understanding of surgery and reduced anxiety
The use of virtual reality to reduce preoperative anxiety in first-time sternotomy patients: a randomized controlled pilot trial (30)	Presurgery	iVR with gaming-type app	sVR with gaming-type app	20	• Significant reduction in anxiety
Virtual reality vs. Kalinox® for management of pain in intensive care unit after cardiac surgery: a randomized study (31)	Postsurgery (during the removal of chest drains)	iVR with relaxing content	Kalinox	200	• Patients in the iVR group reported significantly higher pain levels
Virtual reality and hypnosis for anxiety and pain management in intensive care units: a prospective randomised trial among cardiac surgery patients (32)	Pre–postsurgery	iVR with relaxing content	• Hypnosis only • -Hypnosis + iVR	100	• No significant differences in anxiety or pain between the groups • No significant differences in fatigue, physiological measures, or opioid use

## 5 Tips for the application of VR

Although there is no consensus on the optimal duration of the iVR intervention to produce effective results in patients with ASCVD, it is recommended to consider that prolonged exposure to technology can expose patients to adverse events (33). These adverse effects are described as cybersickness, a type of visually induced motion sickness experienced in virtual environments that causes symptoms such as nausea, dizziness, fatigue, and oculomotor disorders. Cybersickness does not seem to be very common in patients, but it still needs to be taken into account by researchers using validated tools such as the Virtual Reality Symptom Questionnaire (33).

Another aspect to consider is that the proposal of this innovative intervention could create a surprise effect and a strong attraction, conditions that could represent confounding variables to measure the emotional states produced by virtual environments (34). To reduce this risk, it is recommended to implement a familiarization period where, before starting the actual VR treatment, the patient gradually begins to learn about the technology by trying out some scenarios, guided by healthcare professionals (34). If the intervention is planned as a one-off exercise, the familiarization period can be implemented 10 min before the start of the actual operation. However, if multiple intervention sessions are planned, this phase can take place 10 min before the start of the real immersion on the first day of using VR (35) and 5–10 min before the start of the real immersion on the following days (33).

The choice of content to be used for the different purposes is also particularly important. In general, it is recommended to consider using natural scenes, with their own colors, environments, and sounds, because they have a strong distractive capacity (36, 37). Indeed, these can produce a psychophysical state characterized by a reduction of negative emotions and stress, and increased positive emotions, relaxation, calm, and inner peace, bringing to mind positive memories that decrease anxiety and depression (38, 39).

## 6 Implications and further research

In the treatment of ASCVD patients, the use of virtual reality is considered a promising digital health intervention. However, compared with the number of patients who undergo cardiac procedures and surgeries every day and suffer both psychologically and from their side effects, iVR is still rarely used by healthcare professionals. VR seems to be an intervention where the cost–benefit ratio appears favorable, but further research will be needed to confirm this (40).

To ensure the effectiveness and safety of implementing VR in patients with ASCVD, it is important to consider some key factors: (1) prefer the use of iVR over sVR, if possible, to allow patients to interact with the virtual environment more engagingly; (2) consider that, if the objective of the intervention is distraction and relaxation, the use of virtual scenarios inspired mainly by natural environments is preferable to other types of scenarios; (3) limit the immersion time in virtual scenarios to a maximum of 30 min, followed by a 15-min break to reduce the risk of cybersickness; (4) follow a 10-min familiarization phase on the first day of VR use to “acclimate” patients; (5) do a systematic evaluation of potential VR cybersickness after each use of VR through the Virtual Reality Symptom Questionnaire (33).

Future research should focus on several objectives, including: (1) determining the optimal duration of immersion in virtual environments that produces the best results on the psychosomatic axis, considering the impact on mental and physical health of patients with the aim of uniform and standardized application of VR; (2) implementing the creation of virtual content specific to patients with ASCVD to increase awareness and health education and consequently improve various health outcomes specific to these patients; (3) studying the possible side effects of VR, such as cybersickness, and developing strategies to mitigate them; and (4) studying the cost–benefit ratio of VR intervention.

## 7 Conclusions

This state of the art shows that iVR has the potential to be effective in improving physical and psychological outcomes in various cardiologic settings. iVR represents a modern and innovative intervention that can be implemented in healthcare because it is effective, safe, easy to use, and appreciated by patients. It is essential to train healthcare professionals in the use of iVR and create collaborations with technology specialists and patients who can co-design and co-produce virtual scenarios specific to ASCVD patients. More research and fine-tuning of iVR applications are needed to fully realize the benefits of iVR.
